# Parental broad autism phenotype traits and executive function in families of children with autism spectrum disorder

**DOI:** 10.3389/fpsyt.2026.1856417

**Published:** 2026-07-08

**Authors:** Zhijia Li, Yonglu Wang, Shengjian Yin, Chenxi Bao, Hui Fang, Luyang Guan, Fei Wang, Xiaoyan Ke

**Affiliations:** 1 Child Mental Health Research Center, The Affiliated Brain Hospital of Nanjing Medical University, Nanjing, China; 2 The Affiliated Mental Health Center of Jiangnan University, Wuxi Central Rehabilitation Hospital, Wuxi, Jiangsu, China

**Keywords:** autism spectrum disorder (ASD), broad autism phenotype, executive function, parents, psychological paradigm

## Abstract

**Background:**

Parents of children with autism spectrum disorder (ASD) frequently exhibit subclinical Broad Autism Phenotype (BAP) traits, though their relationship with executive function (EF) remains underexplored. This study investigated associations between parental BAP traits and their EF performance.

**Methods:**

The Broad Autism Phenotype Questionnaire (BAPQ) was used to assess the BAP traits in parents of children with ASD and parents of healthy children. Among them 20 of the high-BAP ASD parents with a total BAPQ score exceeding 3.55 and 20 of the parents of typically developing children completed executive function tests. The researchers utilized the flanker, 2-back, and Task-switching paradigm to examine their EF. Correlations analysis was used to analyze BAP-EF relationships.

**Results:**

The self-reported questionnaires indicated that parents of children with ASD had significantly higher BAPQ scores than non-ASD’s parents, with fathers scoring 41.21% versus 15% (χ2 = 31.628, p < 0.01), and mothers 26.25% versus 8.37% (χ2 = 25.764, p < 0.01). Parents of ASD children exhibited significantly prolonged reaction times on the Flanker task (528.95 ± 78.90 ms vs. 426.80 ± 18.40 ms, T = 15.639, p<0.01), with BAPQ total scores positively correlating with slower responses (R² = 0.2325, p = 0.0313). Paradoxically, they demonstrated accelerated 2-Back incongruent reaction times (537.60 ± 80.21 ms vs. 665.70 ± 137.17 ms, T = 6.715, p=0.001) but reduced accuracy (0.947 ± 0.037 vs. 0.983 ± 0.016, T = 16.875, p<0.01), where BAPQ again correlated with reaction times (R² = 0.2318, p = 0.0316). Crucially, these associations were absent in controls, and BAPQ scores showed no relationship with task accuracy in either group.

**Conclusions:**

BAP traits are associated with attentional control challenges (indexed by Flanker Task) and a maladaptive speed-accuracy tradeoffs during working memory demands.

## 1 Introduction

### 1.1 ASD and the broad autism phenotype continuum

Autism Spectrum Disorder (ASD) is a complex neurodevelopmental disorder presented a diverse range of clinical manifestations that can vary significantly among individuals (1). Core diagnostic features encompass persistent deficits in social communication and interaction, alongside restricted, repetitive behavioral patterns (2, 3). Onset typically occurs during infancy or early childhood (4), between the ages of 12 and 36 months, and can persist into adulthood (5). Current epidemiological surveillance by the CDC’s Autism and Developmental Disabilities Monitoring (ADDM) Network identifies ASD prevalence in approximately 3.2% of 8-year-old children (1 in 31), reflecting a substantial public health burden (6). The disorder demonstrates a pronounced male predominance, with a median male-to-female ratio of 4.2:1 (7). Additionally, many affected individuals additionally experience sensory processing abnormalities and comorbid psychiatric conditions including anxiety, depression, and attentional impairments (8, 9).

ASD exhibits high heritability, with twin studies estimating a genetic contribution of 0.83 (95% CI, 0.68∼0.96) (10, 11). Familial recurrence patterns reveal siblings of affected individuals face a 20- to 25-fold increased risk relative to the general population (4). Beyond diagnosed ASD, relatives frequently demonstrate subclinical phenotypic features termed the Broad Autism Phenotype (BAP), characterized by subtle autism-like traits in social reciprocity, communication style, and behavioral flexibility (12, 13). Current data indicate approximately 14–23% of ASD parents exhibit measurable BAP characteristics, significantly exceeding the 5–9% prevalence observed in parents of neurotypical children (14). These traits correlate with observable differences in social interaction, such as atypical gaze patterns and reduced eye contact during interpersonal engagement (15, 16). Furthermore, research on other family members has identified certain personality traits that may be associated with ASD (17). These findings underscore the substantial genetic contribution to the etiology of ASD and highlight the need for further exploration of familial and genetic factors involved in the disorder (18).

### 1.2 Neural substrates of executive dysfunction in autism spectrum disorder

Executive functions (EF), encompassing cognitive control processes like working memory, inhibitory control, and cognitive flexibility, fundamentally underpin adaptive behavior (19, 20). EF difficulties could critically impact parenting efficacy and the relational dynamics within ASD-affected families (21). However, systematic investigation linking quantifiable EF performance with parental BAP traits is notably absent from current research paradigms.

Executive dysfunction represents a core neurocognitive feature in ASD, manifesting as clinically significant impairment (22). Meta-analytic evidence indicates adolescents with ASD exhibit moderate-to-large effect size deficits in EF (23). Functional magnetic resonance imaging (fMRI) during n-back and Set-shifting tasks demonstrates aberrant frontoparietal network in ASD individuals (24, 25). During passive tasks, ASD participants showed significantly delayed N1 latency and heightened P3a amplitude versus health control, indicating altered visual processing and hyper-arousal (26). Aberrant Frontoparietal Network-Somatomotor Network (FPN-SMN) connectivity further correlated with social cognition impairments, confirming triple-network dysregulation as the neural substrate of executive-social deficits in ASD (27, 28). Furthermore, ASD males demonstrated significant Resting-State Functional Connectivity (rsFC) deviations between the ventral attention and default mode networks (VAN-DMN), which were directly associated with deficits in cognitive flexibility and working memory (25).

### 1.3 Transgenerational implications and clinical imperatives

Maternal BAP traits significantly predict impaired social responsiveness in preschool-aged children with ASD, indicating transgenerational transmission of social communication vulnerabilities (29). Fathers demonstrated significantly higher BAP prevalence rates compared to mothers (30), parental BAP profiles permit ASD etiological subgroup identification and generation of targeted parenting interventions (31). While substantial literature examines core BAP characteristics in ASD relatives (32), the neurocognitive correlates of these traits—specifically executive functioning—remain inadequately explored. As a result, the aim of this study is to investigate the executive function of parents exhibiting Broad Autism Phenotype (BAP) traits.

This study addresses this gap by examining EF profiles in ASD parents with confirmed BAP characteristics using standardized neuropsychological tasks. Through assessing the executive function of parents, this research seeks to gain insights into their cognitive profiles and behavioral patterns, particularly in relation to their children diagnosed with ASD. By identifying potential differences in EF, we can better comprehend the implications of BAP traits on parenting practices. This understanding could lead to more tailored interventions and support strategies for families with autistic children. Ultimately, our goal is to enhance the life quality and social adaptability of children with ASD, considering the nuances of parental influence stemming from BAP characteristics.

## 2 Methods

### 2.1 Ethical considerations

This study was approved by the Ethics Committee of the Affiliated Brain Hospital of Nanjing Medical University (Approval Number: 2022-KY022-01), with a clinical trial registration number: ChiCTR2200062132 (registration date:2022/07/24). Written informed consent was obtained from participants after risk-benefit discussions. Research procedures strictly adhered to the 1964 Helsinki Declaration’s ethical framework.

### 2.2 Study procedures and participants

**Figure 1:**
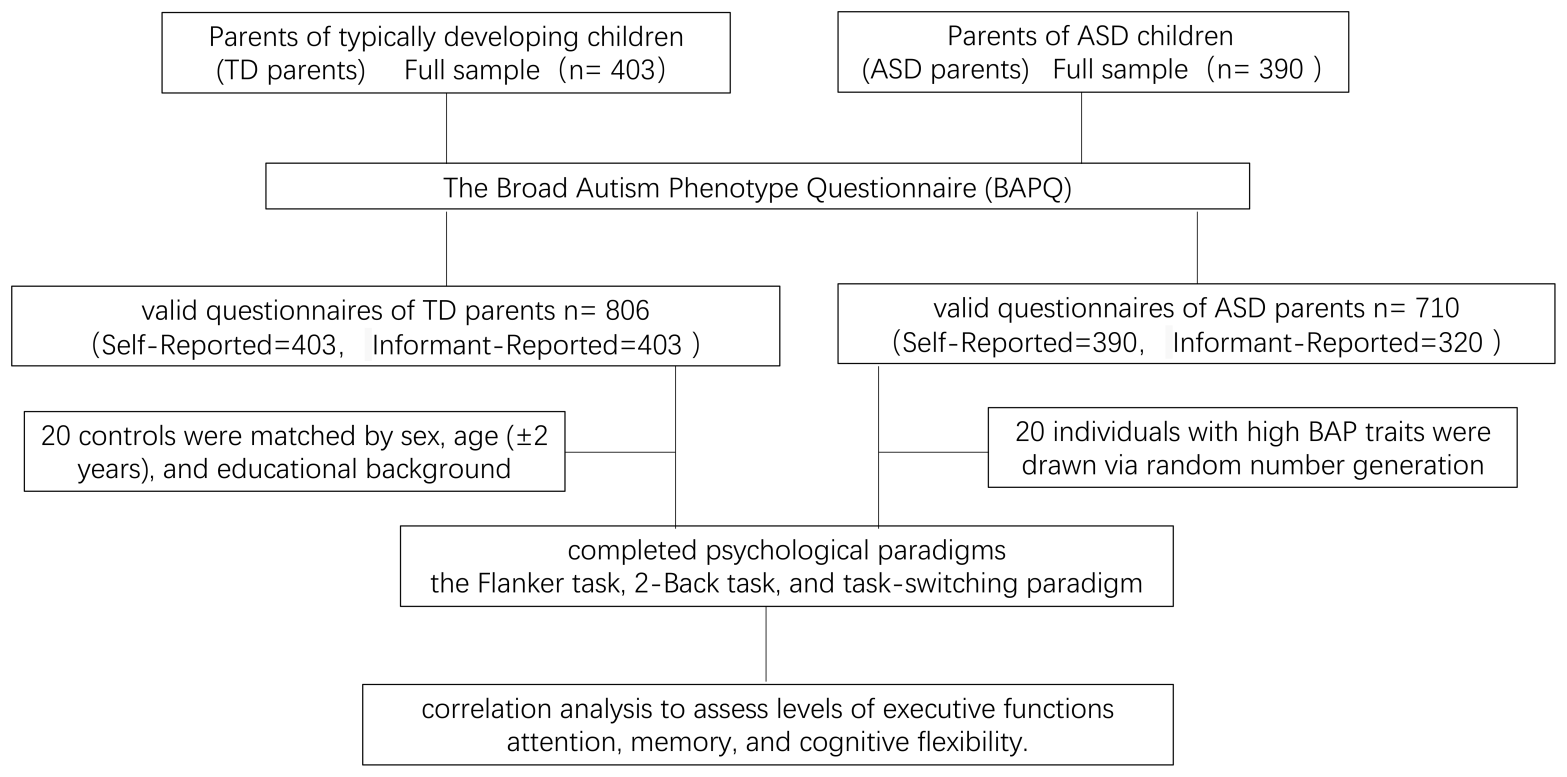
Study procedures and participants. Participant recruitment and executive function assessment workflow. Data were obtained from 390 parents of children with ASD (recruited via clinical cohorts) and 403 parents of typically developing children (community-based controls). A matched subgroup of 40 participants (20 high-BAP/20 controls) completed computerized neurocognitive tasks: flanker task, 2-back task, task-switching paradigm.

The study procedures and participants of this research are illustrated in **Figure 1**. The Broad Autism Phenotype Questionnaire (BAPQ) was used for self-report and respondent report questionnaires (14, 33). A power analysis conducted prior to recruitment using GPower 3.1 indicated that a sample size of 64 participants per group was required. A total of 710 valid questionnaires were collected from parents of children with ASD. Additionally, 806 valid questionnaires were obtained from parents of typically developing children. Twenty participants with high BAP traits parents of children with ASD (total BAPQ scores>3.55) and twenty non-ASD’s parents completed the Flanker, 2-back, and Task-switching paradigm to evaluate their attention, memory, and cognitive flexibility. Performance data were recorded and subsequently analyzed using correlation analysis to explore the relationships between BAP traits and executive function outcomes.

Exclusion criteria for all parents included: personal history of ASD, ADHD, intellectual disability, bipolar disorder, psychotic disorders, or any neurological condition (e.g., epilepsy, traumatic brain injury); current use of psychotropic medications affecting cognition (e.g., benzodiazepines, antipsychotics). Parents with a formal diagnosis of ASD or ADHD were excluded based on a combination of face-to-face clinical interview and self-report questionnaire at recruitment.

## 3 Measures

### 3.1 BAPQ

BAPQ was developed by Hurley (33), which mainly included three aspects: social deficits, stereotyped behavior, and language pragmatics. BAPQ includes three assessment dimensions: Aloofness quantifies, an individual’s tendency toward social aloofness; Pragmatic Language assesses the degree of pragmatic communication deficits; and Rigidity evaluates the extent of cognitive-behavioral rigidity. The scale offers two versions of measurement tools: in this study, the self-report version was utilized for individual self-assessment, while the spousal report version was also employed to reduce response bias. The total score of the BAPQ is calculated as follows: Total Score = 1/36 * ∑ (Aloofness + Pragmatic Language + Rigidity). The established cutoff value is a total score ≥ 3.55 (14), which indicates that the respondent may exhibit significant BAP traits.

### 3.2 Psychological paradigm

#### 3.2.1 Flanker task

**Figure 2:**
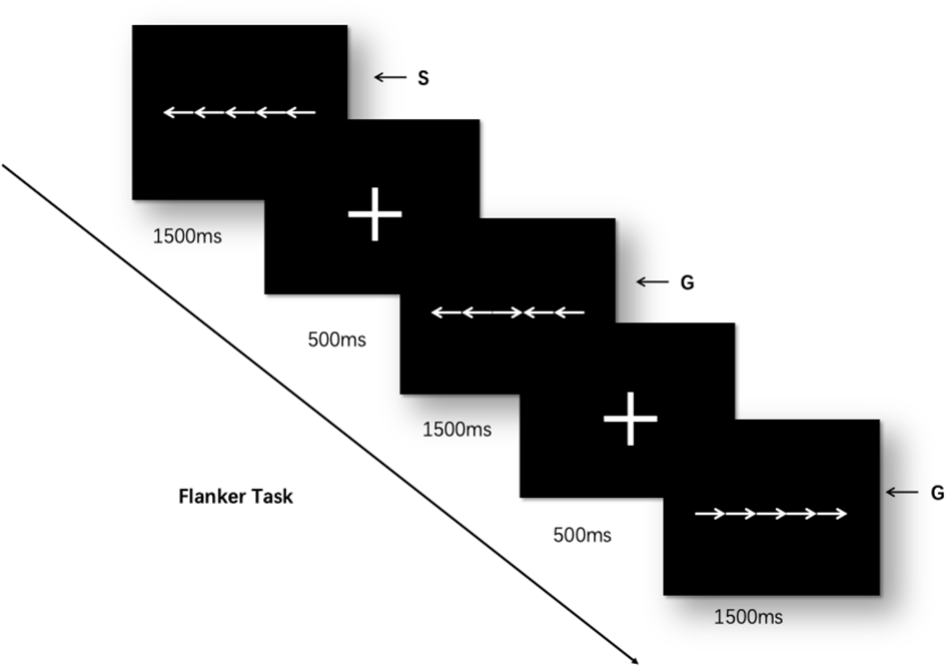
Flanker task procedure. Participants identified the direction of a central target arrow, press “S” for left-facing arrows (← ← ← ← ←), “G” for right-facing arrows (→ → → → → OR ← ← → ← ←), the viewing duration for each image was 1500 ms, with a 500 ms interstimulus interval between trials. A total of 56 trials were conducted, comprising 8 practice trials and 48 test trials.

The Flanker task was utilized to evaluate inhibitory control and attentional capacity among participants (34, 35). In this task (**Figure 2**), subjects viewed a central target arrow surrounded by congruent or incongruent flanker arrows. Participants were required to identify the direction of the central arrow by pressing designated keys promptly and accurately. The task included multiple trials to ensure a robust data set, including 8 practice trials and 48 test trials. All 56 trials reaction times and accuracy were meticulously recorded to assess executive function performance, allowing for the exploration of potential correlations between Broad Autism Phenotype traits and executive functioning in parents of children with ASD. The Flanker task scoring was based on mean reaction time across both congruent and incongruent conditions.

#### 3.2.2 2-back task

**Figure 3:**
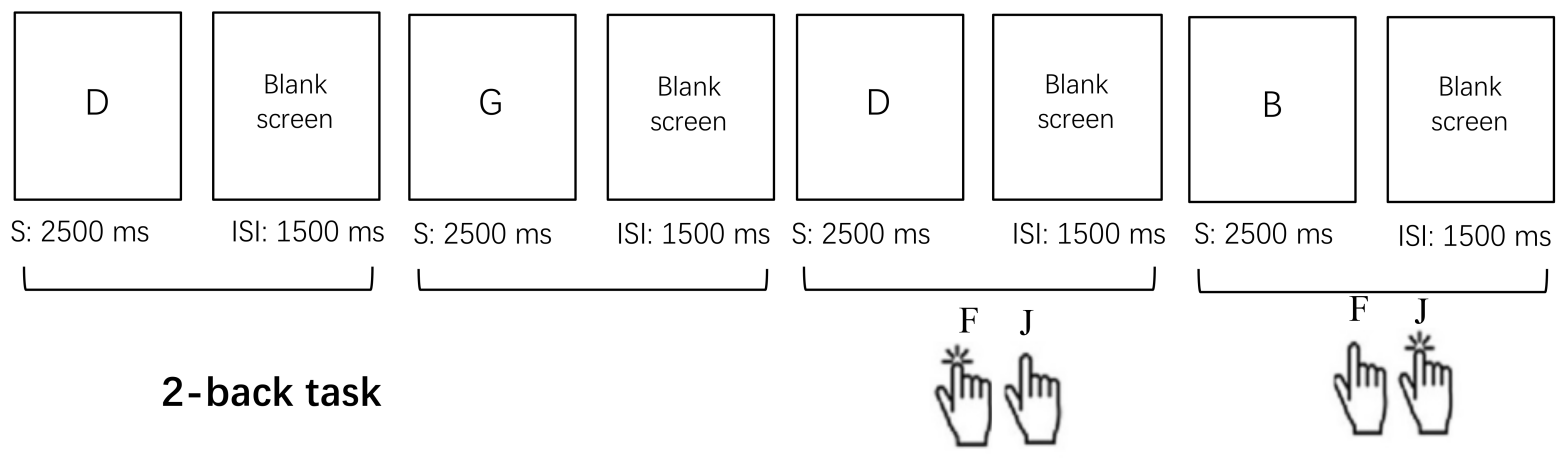
2-back task procedure. In each trial, a sequence of letter stimuli was presented one at a time on a computer screen. The task required participants to determine if the current letter matched the one shown two items prior. If a match occurred, participants pressed the “F” button; if not, they pressed the “J” button. Letters were generated randomly by the software. Total of 50 trials were administered, with accuracy and reaction times recorded to evaluate performance. S, stimulus presentation; ISI, inter-stimulus interval.

The 2-back task was used to assess working memory capacity in participants (36). During the task (**Figure 3**), participants viewed a sequence of letter stimuli presented one at a time on a computer screen. They were required to indicate whether the current stimulus matched the one presented two positions earlier by pressing a designated key. A total of 50 trials were conducted, during which both the accuracy of responses and reaction times were recorded. The task featured a series of randomized stimuli, which aimed to enhance participant engagement and increase the cognitive load challenge. To evaluate participants’ performance effectively, both the rates of correct responses and the corresponding reaction times were meticulously documented throughout the task (35).

#### 3.2.3 Task-switching paradigm

**Figure 4:**
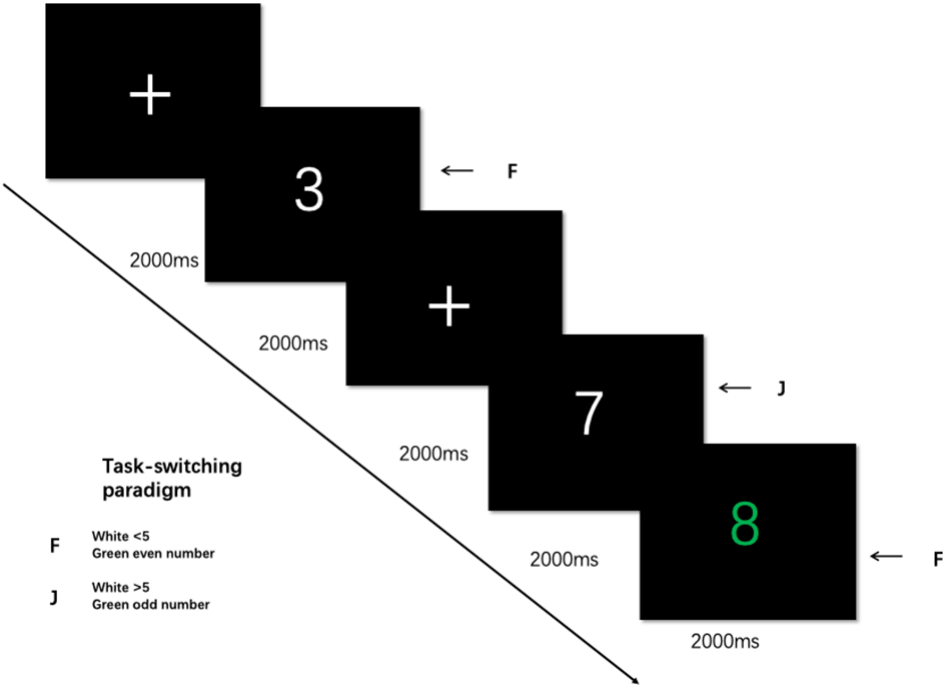
Task-switching paradigm procedure. Participants alternated between two categorization tasks: when numbers were presented in white, they judged the magnitude (pressing ‘F’ for numbers less than 5 and ‘J’ for numbers greater than 5), and when numbers appeared in green, they determined odd/even status (pressing ‘F’ for even numbers and ‘J’ for odd numbers). Each trial included an interstimulus interval of 2000 ms. The procedure comprised 8 practice trials for magnitude judgment, 16 test trials for magnitude judgment, 8 practice trials for odd/even judgment, and 16 test trials for odd/even judgment, 32 mixed trials. Reaction times and accuracy were recorded to measure participants’ cognitive flexibility and adaptability to changing rules.

The task-switching paradigm was implemented to assess in participants’ cognitive flexibility (37). Participants were instructed to alternate between two categorization tasks (**Figure 4**): identifying a series of numbers as either odd or even and determining their magnitude (large or small). Participants were required to assess the magnitude of numbers when they were displayed in white, while they categorized the numbers as odd or even when presented in green. The sequence of tasks included 8 practice trials for magnitude judgment, followed by 16 test trials for magnitude judgment, then 8 practice trials for odd/even judgment, and 16 test trials for odd/even judgment. Finally, participants completed a mixed test comprising 32 trials involving both tasks, 16 trials were switch trials (task changed from the previous trial) and 16 were repeat trials (same task as the previous trial). All responses, including reaction times and accuracy, were meticulously recorded to measure cognitive flexibility and adaptability to changing rules. This assessment aimed to explore the relationship between BAP traits and cognitive flexibility in parents of children diagnosed with ASD (38).

All computerized tasks (Flanker, 2-back, Task-switching) were programmed and presented using E-Prime. Reaction times and accuracy were recorded automatically by the software with millisecond precision.

### 3.3 Data analysis

Data analysis was conducted using appropriate statistical software to assess the relationships between BAP traits and EF measures. Group comparisons were analyzed using independent t-tests or Mann-Whitney U tests, depending on data distribution. Additionally, descriptive statistics were computed to summarize participant performance and scores across Flanker task, 2-back task, and Task-Switching Paradigm. Correlational analyses were performed to examine associations between BAP traits and cognitive performance, with significance levels set at p < 0.05. Linear regression and correlational analyses of BAPQ scores and EF metrics were performed in GraphPad Prism 9.

## 4 Results

### 4.1 BAP traits in parents of children with ASD vs. non-ASD

#### 4.1.1 Self-reported BAP traits

Significant elevations across multiple BAP domains were self-reported by parents of children with ASD versus neurotypical controls. As presented in **Table 1**, mothers demonstrated dramatically higher aloofness (60.34% vs. 31.34%), contrasting with fathers who showed equivalent aloofness levels to controls. Pragmatic language deficits were profoundly elevated in both parental groups: Mothers reported >3-fold higher impairment rates than controls (60.78% vs. 20.28%), while fathers showed nearly triple the prevalence (46.84% vs. 16.13%). Similarly, cognitive rigidity was markedly increased among ASD parents, with mothers reporting >28% prevalence versus 8.76% in controls and fathers showing 26.58% versus 9.68%. Total BAPQ scores confirmed robust group differences: Maternal scores were significantly elevated (32.33% vs. 11.98%), as were paternal scores (41.14% vs. 13.98%). Particularly notable were pragmatic language difficulties, which represented the most prevalent domain.

**Table 1:** Self-reported scores of BAPQ.

Assessment	Parents	Parents of ASD children		Parents of non-ASD’s children		χ^2^	p	Cramér’s V
		N	%	N	%			
Aloofness	Mother	140	60.34	68	31.34	37.946	<0.01^*^	0.291
	Father	24	15.19	28	15.05	0.001	0.972	0.002
Pragmatic Language	Mother	141	60.78	44	20.28	75.913	<0.01^*^	0.411
	Father	74	46.84	30	16.13	38.189	<0.01^*^	0.333
Rigidity	Mother	67	28.87	19	8.76	29.322	<0.01^*^	0.256
	Father	42	26.58	18	9.68	16.954	<0.01^*^	0.222
Total Score	Mother	75	32.33	26	11.98	26.623	<0.01^*^	0.244
	Father	65	41.14	26	13.98	32.393	<0.01^*^	0.307

N, the number of subjects who have reached established cutoff value score ≥ 3.55; %, the percentage of subjects who reached the target. χ2, chi-square value, *P <0.05.

#### 4.1.2 Informant-reported BAP traits in parents

Informant-reported BAPQ data revealed distinct parental profiles in ASD-affected families. Calculated from **Table 2**, Spouse evaluations identified significantly elevated aloofness traits specifically among fathers of children with ASD compared to control fathers (45.27% vs. 32.26%), whereas maternal aloofness showed no significant group difference. This contrasted sharply with self-reported patterns where maternal aloofness was substantially elevated but paternal aloofness remained non-significant. In the pragmatic language domain, informant-reports detected pronounced challenges in both parental groups: ASD mothers exhibited nearly threefold higher impairment rates than controls (40.34% vs. 13.44%), while ASD fathers showed similar significant elevations (34.33% vs. 15.67%). These findings aligned directionally with self-reported data, though self-reports indicated substantially higher maternal prevalence and paternal prevalence. Cognitive rigidity demonstrated no significant differences between groups in informant-ratings for either mothers (8.40% vs. 6.45%) or fathers (2.49% vs. 5.53%). This diverged fundamentally from self-reports where both parents showed dramatically elevated rigidity. Total BAPQ scores confirmed significantly higher pathology in ASD parents through informant-reporting: mothers showed 33.61% prevalence versus 11.83% in controls, while fathers showed 32.84% versus 12.44%. These effect sizes closely paralleled self-reported total scores.

**Table 2:** Informant-reported scores of BAPQ.

Assessment	Parents	Parents of ASD children		Parents of non-ASD’s children		χ^2^	p	Cramér’s V
		N	%	N	%			
Aloofness	Mother	16	13.45	26	13.98	0.017	0.895	0.008
	Father	91	45.27	70	32.26	7.464	0.006^*^	0.134
Pragmatic Language	Mother	48	40.34	25	13.44	28.834	<0.01^*^	0.307
	Father	69	34.33	34	15.67	19.567	<0.01^*^	0.216
Rigidity	Mother	10	8.40	12	6.45	0.413	0.52	0.037
	Father	5	2.49	12	5.53	2.476	0.116	0.077
Total Score	Mother	40	33.61	22	11.83	21.267	0.001^*^	0.264
	Father	66	32.84	27	12.44	17.491	0.001^*^	0.205

N, the number of subjects who have reached established cutoff value score ≥ 3.55; %, the percentage of subjects who reached the target. χ2, chi-square value, *p <0.05.

### 4.2 Executive function assessments across standardized psychological paradigms

Distinct patterns of executive dysfunction emerged in high BAP parents across neurocognitive paradigms. As shown from **Table 3**, flanker task performance revealed significantly prolonged reaction times in the high BAP group (528.95 ± 78.90 ms vs. 426.80 ± 18.40 ms) despite comparable accuracy (0.959 ± 0.027 vs. 0.964 ± 0.033), indicating preserved attentional focus but compromised response inhibition efficiency. In the 2-Back Task, critical deficits surfaced specifically during high-cognitive load conditions. For incongruent trials requiring conflict resolution, the high BAP group showed significantly reduced accuracy (0.947 ± 0.037 vs. 0.983 ± 0.016) coupled with paradoxically accelerated responses (537.60 ± 80.21 ms vs. 665.70 ± 137.17 ms). Total working memory performance approached significance (p=0.067), revealing borderline impairment in information maintenance. Task-switching assessments demonstrated striking context-dependent deficits. During magnitude-judgment trials requiring cognitive flexibility, the high BAP group exhibited significantly slower responses (884.25 ± 454.37 ms vs. 656.90 ± 123.44 ms) despite maintaining accuracy. Mixed judgment trials revealed a clinically noteworthy non-significant trend toward elevated accuracy in the high BAP group, potentially suggesting compensatory cognitive strategies.

**Table 3:** Executive function assessments through psychological paradigms.

Psychological paradigm		Accuracy (mean ± SD)		T	*P* (FDR corrected)	Cohen’s d	Reaction times (mean ± SD)			T	*P* (FDR corrected)	Cohen’s d
		High BAP	Non-ASD				High BAP		Non-ASD			
Flanker Task	Flanker Task	0.959 ± 0.027	0.964 ± 0.033	1.319	0.603	0.166	528.95 ± 78.90	426.80 ± 18.40		15.639	0.00007*	1.783
2-Back Task	2-Back Total	0.957 ± 0.036	0.975 ± 0.022	1.931	0.224	0.604	515.10 ± 76.94	674.25 ± 140.06		6.807	0.000245*	1.408
	2-Back Congruent	0.9636 ± 0.050	0.966 ± 0.040	0.338	0.868	0.053	496.15 ± 74.61	682.65 ± 176.39		17.193	0.000467*	1.377
	2-Back Incongruent	0.947 ± 0.037	0.983 ± 0.016	16.875	0.002*	1.263	537.60 ± 80.21	665.70 ± 137.17		6.715	0.00175*	1.14
Task-switching	Magnitude judgment	0.975 ± 0.065	0.988 ± 0.038	2.408	0.623	0.244	884.25 ± 454.37	656.90 ± 123.44		12.079	0.056	0.683
	Odd/even judgment	0.994 ± 0.019	0.984 ± 0.034	4.968	0.452	0.363	604.35 ± 150.28	593.90 ± 87.52		1.086	0.79	0.085
	Mixed judgement	0.931 ± 0.086	0.881 ± 0.123	0.101	0.338	0.471	826.35 ± 218.10	800.85 ± 145.96		5.085	0.782	0.137

Colored values*:p<0.05 after Benjamini–Hochberg FDR correction

### 4.3 Correlation of BAPQ scores with executive function deficits

**Figure 5:**
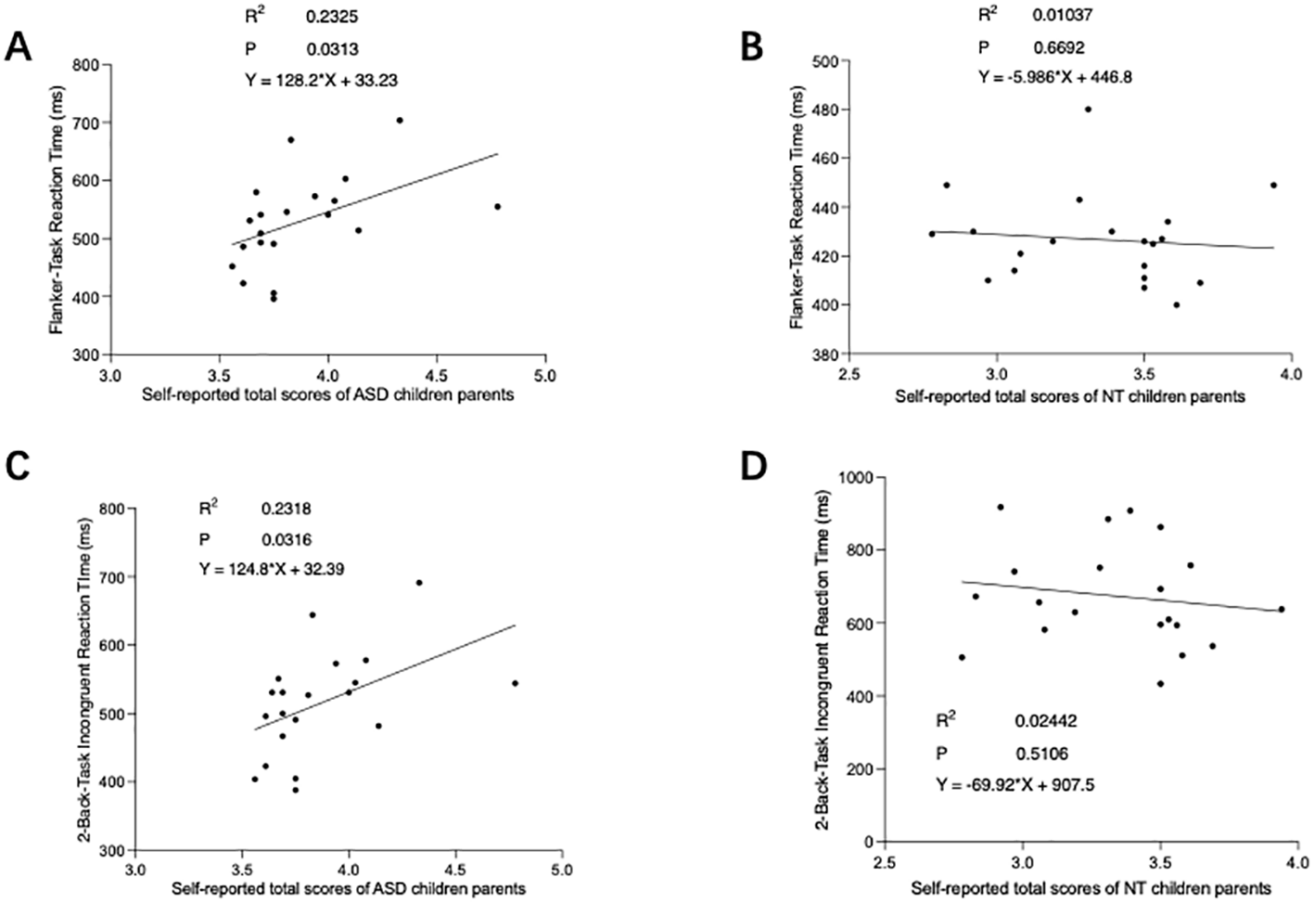
Correlation between BAPQ scores and executive function performance. **(A)** correlation between BAPQ self-reported total scores and flanker-task reaction time of ASD children’s parents. **(B)** Correlation between BAPQ self-reported total scores and flanker-task reaction time of NT children’s parents. **(C)** Correlation between BAPQ self-reported total scores and 2-back-task incongruent reaction time of ASD children’s parents. **(D)** Correlation between BAPQ self-reported total scores and 2-back-task incongruent reaction time of NT children’s parents. BAPQ, the broad autism phenotype questionnaire; NT, neurotypical children’ s parents.

Parents of children with ASD exhibited significantly longer reaction times on the Flanker task compared to parents of neurotypical children (**Table 3**). As illustrated in **Figure 5A**, this deficit in inhibitory control was positively correlated with total BAPQ scores (R² = 0.2325, p = 0.0313), suggesting that BAP traits contribute to attentional conflict resolution difficulties. Although aloofness and pragmatic language subscales showed positive trends with Flanker reaction times (**Supplementary Figures 1A–C**, p = 0.0671 and p = 0.0975 respectively), these relationships fell below statistical significance. No correlations existed between BAPQ and Flanker performance in parents of neurotypical children (**Figure 5B**; **Supplementary Figures 1D–F**).

Conversely, ASD parents demonstrated paradoxically faster reaction times on the 2-Back incongruent condition compared to controls but achieved lower accuracy rates (**Table 3**). Total BAPQ scores positively correlated with reaction times in ASD parents (**Figure 5C**; R² = 0.2318, p = 0.0316), while aloofness and pragmatic language subscales showed non-significant positive trends (**Supplementary Figures 2A-C**). Again, no BAPQ correlations emerged in the control group across any measures (**Figure 5D**; **Supplementary Figures 2B–D**), and BAPQ scores showed no relationship with 2-Back accuracy in either group (**Supplementary Figure 3**).

## 5 Discussion

This study represents the first investigation into the neurocognitive correlates of BAP traits among parents of children with ASD. Our findings reveal significant EF challenges in high-BAP parents that correspond with distinct patterns of self-reported and informant-rated BAP characteristics. These results demonstrating that EF challenges—particularly in processing speed, inhibitory control, and cognitive flexibility—underlie core BAP traits. This finding aligns with the procedural deficit hypothesis of ASD (39, 40), suggesting that slowed information processing compromises social communication efficiency—a core dimension of BAP (41, 42). The prolonged Flanker-task reaction times and their correlation with BAPQ scores reflect inefficient conflict monitoring (43), likely stemming from altered connectivity between the anterior cingulate cortex (ACC) and dorsolateral prefrontal cortex (dlPFC) (44). Genetic factors associated with ASD disrupt GABAergic inhibition in front-striatal circuits, such as the loss of CNTNAP2 function (45), compromising the suppression of irrelevant stimuli during attention tasks (46). This deficit is amplified in individuals with high BAPQ scores due to neural inefficiency in conflict resolution networks.

The paradoxically faster 2-Back reaction times coupled with reduced accuracy in parents of individuals with ASD indicate a maladaptive compensatory strategy As in Baddeley’s model of working memory (47). When cognitive load increases, individuals with BAP traits often sacrifice precision for speed due to working memory capacity limitations (48). For example, in driving simulation experiments, the introduction of additional working memory load (such as simultaneously processing visual and memory tasks) increases cognitive burden, leading drivers to reduce the number of precise control actions to prioritize time-sensitive demands, ultimately resulting in an overall decline in driving performance. This indicates that individuals under high load will sacrifice accuracy for operational speed to align with the constraints of working memory resources (49).

Additionally, The paradoxical combination of faster responses during high-conflict 2-Back trials coupled with reduced accuracy indicates an impulsive decision-making style potentially reflecting diminished social motivation, as conceptualized in the social motivation theory of ASD (50, 51), supporting the cognitive inflexibility framework central to ASD pathophysiology (52). Critically, the marked dissociation between informant-reported and self-perceived aloofness—significant paternal deficits detected only through spouse-ratings—highlights methodological considerations for BAP assessment that parallel insight deficits observed in ASD (53).

The EF-BAP relationships observed suggest that subclinical ASD manifestations exist along a neurocognitive continuum. Parents exhibiting BAP characteristics demonstrate attenuated forms of executive dysfunction classically associated with ASD, particularly in front-striatal circuitry supporting response inhibition and cognitive flexibility (15, 54). The preserved accuracy during simpler EF tasks alongside significant processing speed deficits indicates compensatory neurocognitive mechanisms—potentially reflecting increased recruitment of parietal regions during executive control tasks as identified in neuroimaging studies of BAP traits (50, 55, 56). The gender-specific EF patterns (greater maternal processing speed deficits but comparable paternal accuracy challenges) parallel differential expression of ASD-related genes on sex chromosomes (57), suggesting sexually dimorphic pathways in BAP neurocognition that warrant further investigation. Our findings collectively indicate that executive dysfunction constitutes a core component of the BAP, potentially be associated with the generational transmission of autistic traits through compromised parental scaffolding and social learning opportunities. In addition, the specificity of the BAP-EF relationship – observed only in ASD parents and not in controls – supports the notion that BAP traits represent a familial endophenotype. This distinction strengthens the construct validity of BAP as a meaningful subclinical phenotype. Furthermore, Self-reports, but not informant reports, detected significant group differences in rigidity. Three potential explanations may account for this discrepancy: (1) rigidity may be context−dependent and more evident outside the home; (2) long−term cohabitation may normalize mild rigid behaviors in the eyes of spouses; and (3) rigidity has internal cognitive components that are less observable to others.

Several methodological limitations merit consideration. First, our sample predominantly comprised Chinese parents, limiting cross-cultural generalizability—future multicenter studies should examine BAP-EF relationships across diverse ethnic populations as cultural factors influence autism manifestation dynamics. Besides, an additional limitation of this study is the limited sample size for EF assessments. Then, our cross-sectional design precludes causal interpretations regarding BAP-EF relationships; longitudinal cohort studies examining developmental trajectories would better establish directional pathways. In addition, future research should incorporate functional neuroimaging to identify neural substrates of observed EF-BAP relationships and examine gene & cognition interactions through polygenic scoring approaches. What’s more, this study did not formally assess or control for parental intellectual ability (IQ), which is known to be closely associated with executive function performance, particularly processing speed and working memory (58). Future studies should include an estimated IQ measure to rule out potential confounding effects of general cognitive ability on the observed BAP−EF relationships.

In conclusion, this investigation substantiates that executive dysfunction represents an integral component of the BAP, characterized by domain-specific deficits that align with core autistic traits. The observed EF profiles—particularly slowed processing speed and response inhibition deficits—appear implicated in social-communicative BAP manifestations. These findings illuminate previously unrecognized neurocognitive dimensions of the familial autism phenotype while providing critical insights regarding neurodevelopmental mechanisms that warrant clinical consideration when delivering family-centered ASD interventions. Future research should establish whether targeted EF training might beneficially modulate BAP expression or parenting efficacy in ASD-affected families.

## Data Availability

The original contributions presented in the study are included in the article/**Supplementary Material**. Further inquiries can be directed to the corresponding authors.

## Supplementary material

Supplementary table 1 Comparison of parental sex distribution.

Supplementary Table 2 Comparison of parental age distribution.

Supplementary Table 3 Comparison of parental education background distribution.

Supplementary Figure 1 Correlation between BAPQ scores and flanker-task reaction time. **(A–C)** Correlation between BAPQ self-reported scores [**(A)** aloofness, **(B)** pragmatic language, **(C)** rigidity] and Flanker-Task reaction time of ASD children’s parents. **(D–F)** Correlation between BAPQ self-reported scores [**(D)** aloofness, **(E)** pragmatic language, **(F)** rigidity] and Flanker-Task reaction time of NT children’s parents.

Supplementary Figure 2 Correlation between BAPQ scores and 2-back-task incongruent reaction time. **(A–C)** Correlation between BAPQ self-reported scores [**(A)** aloofness, **(B)** pragmatic language, **(C)** rigidity] and 2-Back-Task Incongruent reaction time of ASD children’s parents. **(D–F)** Correlation between BAPQ self-reported scores [**(D)** aloofness, **(E)** pragmatic language, **(F)** rigidity] and 2-Back-Task Incongruent reaction time of NT children’s parents.

Supplementary Figure 3 Correlation between BAPQ scores and 2-back-task incongruent accuracy. **(A–D)** Correlation between BAPQ self-reported scores [**(A)** total, **(B)** aloofness, **(C)** pragmatic language, **(D)** rigidity] and 2-Back-Task accuracy of ASD children’s parents. **(D–F)** Correlation between BAPQ self-reported scores [**(E)** total, **(F)** aloofness, **(G)** pragmatic language, **(H)** rigidity] and 2-Back-Task Incongruent accuracy of NT children’s parents.
