# Supplementary material for: Parental broad autism phenotype traits and executive function in families of children with autism spectrum disorder
Source: Front Psychiatry. 2026 Jul 8;17:1856417. doi: 10.3389/fpsyt.2026.1856417 (PMC13389495; doi:10.3389/fpsyt.2026.1856417)
Supplement: Supplementary table 1 — Comparison of parental sex distribution. [file DataSheet1.docx]

**Supplementary Tables**

**Supplementary Table S1. Comparison of Parental Sex Distribution**

| **Parents** | **Parents of ASD children** | **Parents of non-ASD’s children** | **χ2** | **p** |
| --- | --- | --- | --- | --- |
| **Mother** | 232 | 217 | 2，568 | 0.109 |
| **Father** | 158 | 186 |  |  |

Note. χ2= chi-square value

**Supplementary Table S2. Comparison of Parental Age Distribution**

| **Parents of ASD children**  **(mean ± SD)** | **Parents of non-ASD’s children**  **(mean ± SD)** | **T** | **p** |
| --- | --- | --- | --- |
|  |  |  |  |
| 39.69±6.225 | 40.36±5.853 | 0.102 | 0.749 |

**Supplementary Table S3. Comparison of Parental Education Background Distribution**

| **Parents of ASD children** | | **Parents of non-ASD’s children** | | **χ^2^** | **p** |
| --- | --- | --- | --- | --- | --- |
| **Education Background** | **N** | **Education Background** | **N** |  |  |
| Junior school  High school  Undergraduate  Graduate | 112  154  109  15 | Junior school  High school  Undergraduate  Graduate | 119  162  112  10 | 1.243 | 0.743 |

Note. χ2= chi-square value

**Supplementary Figures**

**Supplementary Figure S1. Correlation Between BAPQ Scores and Flanker-Task reaction time.** A-C. Correlation between BAPQ self-reported scores (A: aloofness, B: pragmatic language, C: rigidity) and Flanker-Task reaction time of ASD children’s parents. D-F. Correlation between BAPQ self-reported scores (D: aloofness, E: pragmatic language, F: rigidity) and Flanker-Task reaction time of NT children’s parents.

**
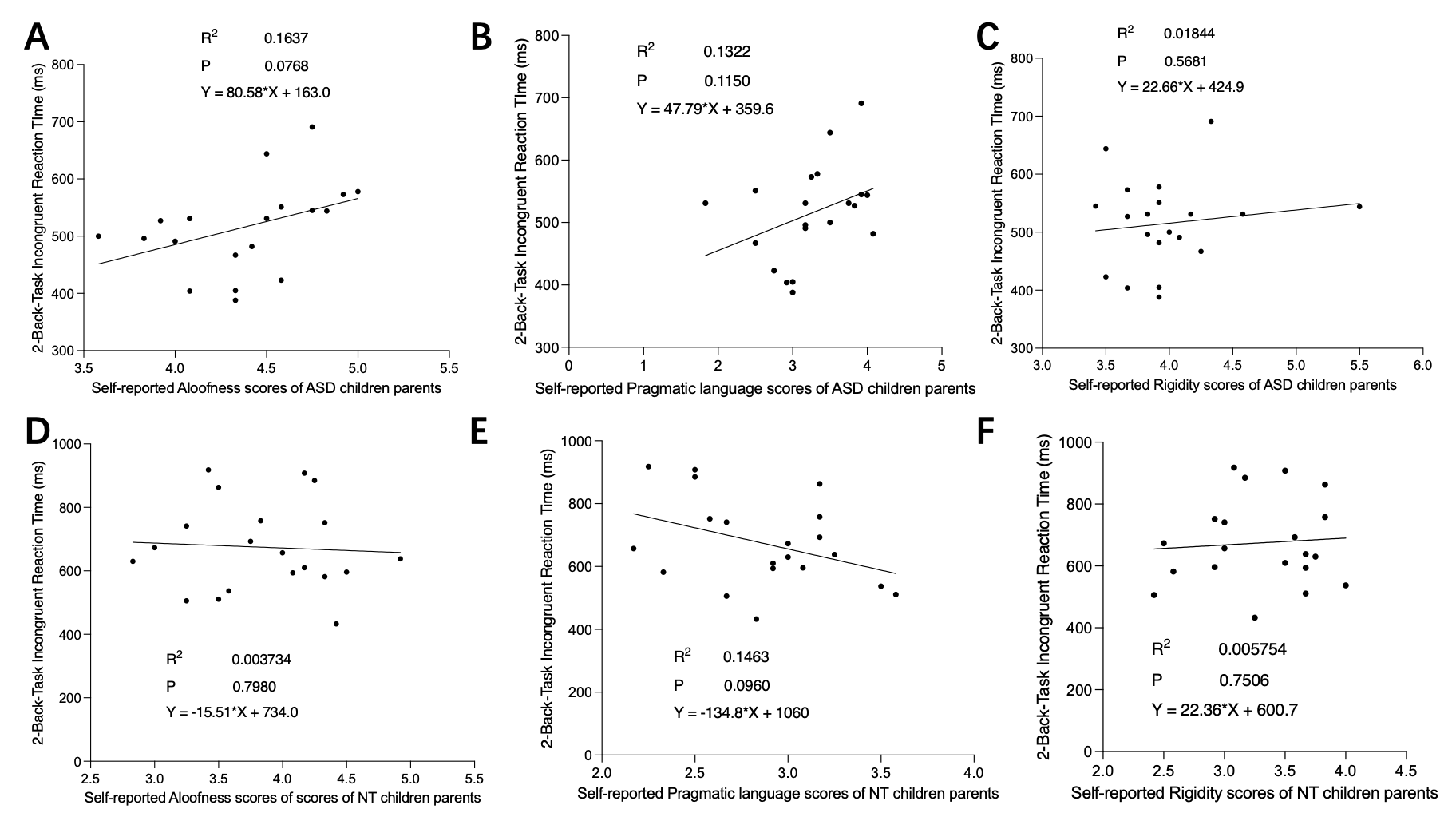
**

**Supplementary Figure S2. Correlation Between BAPQ Scores and** **2-Back-Task Incongruent Reaction Time.** A-C. Correlation between BAPQ self-reported scores (A: aloofness, B: pragmatic language, C: rigidity) and 2-Back-Task Incongruent reaction time of ASD children’s parents. D-F. Correlation between BAPQ self-reported scores (D: aloofness, E: pragmatic language, F: rigidity) and 2-Back-Task Incongruent reaction time of NT children’s parents.

**Supplementary Figure S3. Correlation Between BAPQ Scores and 2-Back-Task Incongruent Accuracy.** A-D. Correlation between BAPQ self-reported scores (A: total, B: aloofness, C: pragmatic language, D: rigidity) and 2-Back-Task accuracy of ASD children’s parents. D-F. Correlation between BAPQ self-reported scores (E: total, F: aloofness, G: pragmatic language, H: rigidity) and 2-Back-Task Incongruent accuracy of NT children’s parents.
